# Screening of the spine in adolescents: inter- and intra-rater reliability and measurement error of commonly used clinical tests

**DOI:** 10.1186/1471-2474-15-37

**Published:** 2014-02-10

**Authors:** Ellen Aartun, Anna Degerfalk, Linn Kentsdotter, Lise Hestbaek

**Affiliations:** 1 Department of Sports Science and Clinical Biomechanics, Faculty of Health Sciences, University of Southern Denmark, Campusvej 55, DK-5230 Odense M, Denmark; 2 Nordic Institute of Chiropractic and Clinical Biomechanics, Campusvej 55, DK-5230 Odense M, Denmark; 3 Clinical practice, Ryggcentrum Lund, Botulfsgatan 2, SE-22350 Lund, Sweden; 4 Clinical practice, Aktivera kiropraktik & rehab, Glimmervägen 14, SE-19131 Sollentuna, Sweden

**Keywords:** Reliability, Measurement error, Scoliosis, Hypermobility, Intersegmental mobility, Spine, Adolescents, Mobility

## Abstract

**Background:**

Evidence on the reliability of clinical tests used for the spinal screening of children and adolescents is currently lacking. The aim of this study was to determine the inter- and intra-rater reliability and measurement error of clinical tests commonly used when screening young spines.

**Methods:**

Two experienced chiropractors independently assessed 111 adolescents aged 12–14 years who were recruited from a primary school in Denmark. A standardised examination protocol was used to test inter-rater reliability including tests for scoliosis, hypermobility, general mobility, inter-segmental mobility and end range pain in the spine. Seventy-five of the 111 subjects were re-examined after one to four hours to test intra-rater reliability. Percentage agreement and Cohen’s Kappa were calculated for binary variables, and interclass correlation (ICC) and Bland-Altman plots with Limits of Agreement (LoA) were calculated for continuous measures.

**Results:**

Inter-rater percentage agreement for binary data ranged from 59.5% to 100%. Kappa ranged from 0.06-1.00. Kappa ≥ 0.40 was seen for elbow, thumb, fifth finger and trunk/hip flexion hypermobility, pain response in inter-segmental mobility and end range pain in lumbar flexion and extension. For continuous data, ICCs ranged from 0.40-0.95. Only forward flexion as measured by finger-to-floor distance reached an acceptable ICC(≥ 0.75). Overall, results for intra-rater reliability were better than for inter-rater reliability but for both components, the LoA were quite wide compared with the range of assessments.

**Conclusion:**

Some clinical tests showed good, and some tests poor, reliability when applied in a spinal screening of adolescents. The results could probably be improved by additional training and further test standardization. This is the first step in evaluating the value of these tests for the spinal screening of adolescents. Future research should determine the association between these tests and current and/or future neck and back pain.

## Background

Spinal pain in children and adolescents is common. Research in the last decades has shown that spinal pain starts early in life and that prevalence rates increase rapidly during adolescence, reaching adult levels around the age of 18 [1,2]. Furthermore, spinal pain has a tendency to continue and an early onset of back pain is found to increase the risk of poor spinal health later in life [3-5], with all its well-known consequences, including very high societal costs [6,7]. Therefore, developing preventive strategies for spinal pain is highly desirable [8,9].

To develop *targeted* preventive strategies, it is necessary to determine the measures that can identify and predict spinal pain in children and adolescents. Currently, the spine of children and adolescents is often screened by medical doctors, nurses, physiotherapists or chiropractors in schools and in clinics involving several commonly used tests and measurements. If clinical tests are used to evaluate children or adolescent’s spine for current or future spinal pain, it is of vital importance that these tests are reliable in a normal population. Some of these tests have been tested for reliability in earlier studies [10-20]. However, they have typically been tested in adults and based on clinical populations rather than normal populations. The reliability of tests may vary between clinical and normal populations simply because of the heterogeneity found in the normal population. In a normal population we can expect a low prevalence of the tests; this is likely to result in low reliability because the lower prevalence of positive findings gives a large influence of Kappa values in the case of disagreement. Moreover, Kappa values cannot be compared when the prevalence rates vary [21]. Therefore, reliability measured in a clinical setting cannot be extrapolated to a screening setting, and thus, the reliability of spinal tests in a screening setting is still unclear. Moreover, the reliability of these tests when applied to children and adolescents is unknown.

Therefore, the purpose of this study was to determine the inter- and intra-rater reliability and measurement error of commonly used clinical tests when screening the spine in adolescents including tests for scoliosis, hypermobility, general mobility with end range pain and inter-segmental mobility of the spine.

## Methods

### Design

An inter- and intra-rater reliability study.

### Subjects

We recruited pupils from the 6^th^ and 7^th^ grades (12–14 years of age) from a school on the island of Funen in Denmark. There were no exclusion criteria. Our aim was to include 100 participants for inter-rater reliability testing, of which 70 were planned to be re-examined in order to test intra-rater reliability. Therefore, we enrolled five classes in the project: one 6th grade and four 7th grade classes.

### Raters and standardisation session

The raters were two chiropractors, both with nine years of clinical experience. To practise the examination procedure and to improve the homogeneity between raters, a standardisation session was held one week before the beginning of the study. The raters practised the sequence of tests in the examination protocol, performing the different tests and measurements and, on the basis of their interpretation, consensus about the procedure was established.

### Procedure

The study was carried out over 4 days spread over a 2-week period in January and February 2012. Subjects were informed that they would have their spine examined as part of a study investigating adolescents and back pain. During the screening, the two raters were placed in separate rooms and blinded to each other’s results. The students were distributed between the two raters in random order. Each subject was examined by both chiropractors, enabling the assessment of inter-rater reliability. For analysing of the intra-rater variability for the two raters, we selected a random sample for a second examination and equally distributed the sample between the two raters. The second examination was performed one to 4 hours later to minimise the risk of change in the subject’s biomechanical state. One hour between the examinations was considered to be sufficient to limit examiner recall because there were many tests and about 20 subjects in between the first and second assessment. Furthermore, no extensive pain provocation was induced during the tests and thus, a longer time period was not needed for recovery. Subjects were examined with clothes on and shoes off. Each examination was limited to 4 minutes to mimic the time likely to be available for screening purposes. All tests were performed as single measures and raters were only allowed to communicate with the subjects for instructional purposes and to ask if pain was present, where relevant. Both raters were observed by two graduate chiropractic students in order to detect and correct possible deviations from the protocol and to note test duration. The observers also switched between the raters to detect any discrepancies in the performance of the tests and the interpretations of the results.

### Test variables

We selected tests that are commonly used by health care workers in a screening setting. The clinical tests assessed were the following:

Assessment of scoliosis

– Shoulder height difference (binary)

– Adam’s Forward Bend Test (binary)

Assessment of hypermobility

– Knee extension (binary)

– Elbow extension (binary)

– Thumb abduction/opposition with wrist flexion (binary)

– Fifth finger extension (binary)

– Trunk/hip flexion (binary)

– Generalized hypermobility was estimated with the Beighton score [22] where the number of positive tests were summed with a maximum score of 9. This score was dichotomized, first in a variable with a cut-off point ≥4 and then in a variable with a cut-off point ≥5 as performed in another study [14].

Assessment of spinal mobility

– Forward flexion finger-floor-distance (FFD) (continuous)

– Lateral flexion FFD (continuous)

– The Schober test (continuous)

Assessment of inter-segmental mobility

– Restriction (binary)

– Pain response (binary)

End range pain on active range of motion

– Pain in maximal lumbar flexion, extension and lateral flexion (binary)

– Pain in maximal cervical flexion, extension and rotation (binary)

For a detailed description of these tests, see Appendix 1.

### Data analyses

All data were written on paper sheets during the test session. Then, all collected data were entered twice into EpiData by two assistants in order to eliminate entry errors and disagreement in the interpretation of the paper sheets. All analyses were performed using STATA version 11.2 (StataCorp LP, College Station, TX, USA). We have used the definition from the COSMIN study, where the overall domain reliability is defined as “the degree to which the measurement is free from measurement error” [23] and aimed to estimate this.

For *binary variables,* the total percentage of agreement (P_a_) was calculated. Kappa with 95% confidence intervals (CI) was used to assess the proportion of agreement beyond that expected by chance [24]. To interpret the strength of reliability within the Kappa values, we used the classification by Landis and Koch [25]: <0.00, poor; 0.00-0.20, slight; 0.21-0.40, fair; 0.41-0.60, moderate; 0.61-0.80, substantial; 0.81-1.00, almost perfect. In this study, a score of 0.40 or higher was considered clinically acceptable. This cut-off score has also been used in other studies on reliability of spinal examination procedures [19,26].

In the interpretation, the prevalence of positive findings was taken into account because the lower prevalence of positive findings gives a large influence of Kappa values, resulting in imprecise estimates of the Kappa values in case of disagreement. Furthermore, the sample size in our study might result in very small cell sizes which will hamper the stability of the Kappa statistics even more.

For *continuous variables*, ICC with 95% CI was used for assessing reliability. A general definition of the ICC is that it expresses the ratio of the variance between subjects to the total variance [26]. We used the two-way random effects model ICC [2.1] for single ratings [26]. Measurement error was visualised using Bland-Altman plots [27]. The mean of the scores for the paired measures for each subject was plotted against the differences (d) between these two measurements. The plots were inspected visually for signs of heteroscedasticity in terms of increasing random error with higher measurement values. The 95% limits of agreement (LoA) were calculated from the mean of the differences (*đ*) : (*đ*+/-1.96 × SD_difference_). LoA cover both systematic and random differences between two observers by quantifying the range of values that can be expected to cover 95% of their differences [28]. As a measure of the random error of a single measurement by a single rater, the standard error of measurement (SEoM) was calculated using the square root of the error variance from the two-way ANOVA random effect model. An ICC score of 0.75 or higher has been suggested as good reliability [29], classified as good in other studies of reliability [20,30] and was also considered clinically acceptable in our study.

Since the definitions of clinically acceptable Kappa and ICC values are both arbitrary and the Kappa statistics is very sensitive to the number of positive findings, the interpretation of the reliability of the tests will be based on all parameters in the analyses, including percentage agreement, Kappa/ICC values, measurement error and limits of agreement.

The intra-rater reliability and measurement error were calculated and reported separately for the two raters.

### Ethics

One week prior the study, the parents of the involved students, received a letter including information about the project and a form to refuse participation of their child. Thus, if they did not return the non-consent form, it was considered as passive consent. This form of passive consent was reviewed by the Regional Committee for Health Research Ethics with the rest of the project protocol. The conclusion was that the project was acceptable according to Danish legislation and did not require formal approval because all tests were non-invasive and there were no physical interventions involved [31]. The study is registered in the Danish Data Protection Agency (Reference number: 2010-41-5147). Prior to this study, we conducted a pilot test with two school classes in order to select the feasible tests.

## Results

There were 116 pupils in the five enrolled classes and 111 participated in the inter-rater reliability study, resulting in a participation rate of 95.7%. Not one pupil refused participation, but five pupils were absent from school on the day of screening. In the study population, the boys represented 53.2% (n = 59), and 23.4% (n = 26) and 76.6% (n = 85) represented 6^th^ and 7^th^ grade pupils respectively. In the intra-rater reliability study, 75 of the 111 pupils participated. All examinations were completed within the upper time limit of 4 minutes.

The double entry of the data detected just a few errors (<0.1%) which were corrected before the analyses.

### Deviations from the standardised procedure

a) The first four subjects were examined with their shoes on by Rater 2. This could have led to a misleading poor reliability and/or measurement error for the assessments of spinal mobility. Therefore, we were interested to calculate the variation after we removed data on these subjects. These calculations affected the results at the second decimal point in ICC.

b) When the data were sampled and analysed, we identified a discrepancy in the performance of the Schober test, where Rater 1 had correctly been rounding up or down to the nearest half centimeter, while Rater 2 had only used whole centimeter measures. This could have contributed to an erroneous inter-rater reliability and measurement error of the Schober test in our study. Therefore, we decided to not report these results.

### Inter-rater reliability

Among the 18 binary tests with cell sizes above five, we reached a Kappa value ≥ 0.4 in 10 (see Table 1). The percentage agreement was ≥ 0.85 in 18 of the 27 tests. Based on Kappa values alone, the assessments for scoliosis were not reliable, with *K* = 0.20 (95% CI: 0.10 - 0.28) for shoulder height difference and *K* = 0.32 (95% CI: 0.04 - 0.60) for the Adam’s Forward Bend Test. Reliability for hypermobility ranged between *K* = 0.12 (95% CI: -0.15 - 0.38) for the right knee and *K* = 1.00 (95% CI: 1.00 - 1.00) for trunk flexion. Assessing inter-segmental mobility in order to detect restriction resulted in Kappa values ranging from *K* = 0.06 (95% CI:-0.08 - 0.19) in the cervical spine to *K* = 0.25 (95% CI: 0.07 - 0.43) in the lumbar spine. The reliability of inter-segmental mobility with pain response ranged from *K* = 0.45 (95% CI: 0.26 - 0.64) in the thoracic spine to *K* = 0.69 (95% CI: 0.54 - 0.84) in the lumbar spine. For end range pain, the reliability ranged between *K* = 0.22 (95% CI: -0.18 - 0.63) for neck extension, and K = 0.59 (95% CI: 0.27 - 0.92) for lumbar flexion. Tests with small cell sizes should be interpreted with caution and is marked with a star in Table 1.

**Table 1:** Inter-rater reliability of commonly used clinical tests of the spine (binary variables)

**Test (missing values)**		**Prevalence of positive findings**		**Agreement**	**Reliability**
		**Rater 1**	**Rater 2**		
		**% (n)**	**% (n)**	**P**_**a **_**(%)**	**Kappa (95% CI)**
**Scoliosis**					
Shoulder height		32.4 (36)	35.1 (39)	59.5	0.20 (0.10, 0.28)
Adam’s forward bend test (1)		11.7 (13)	7.3 (8)	88.2	0.32 (0.04, 0.60)
**Hypermobility**					
Knee	R	2.7 (3)	9.0 (10)	90.1	0.12 (-0.15, 0.38)
	L	1.8 (2)	3.6 (4)	96.4	0.32 (-0.17, 0.81)*
Elbow	R	18.0 (20)	17.1 (19)	88.3	**0.60 (0.40, 0.79)**
	L	22.5 (25)	24.3 (27)	82.0	**0.50** (0.31, 0.69)
Thumb	R	9.9 (11)	9.9 (11)	94.6	**0.70 (0.47, 0.93)**
	L	12.6 (14)	7.2 (8)	94.6	**0.70 (0.48, 0.92)**
Fifth finger	R	2.7 (3)	0.9 (1)	98.2	**0.49** (-0.11, 1.00)*
	L	3.6 (4)	0.9 (1)	97.3	0.39 (-0.15, 0.93)*
Trunk flexion (3)		1.8 (2)	1.8 (2)	100.0	1.00 (1.00, 1.00)*
Beighton score ≥ 4		6.3 (7)	4.5 (5)	96.4	**0.65** (0.33, 0.97)
Beighton score ≥ 5		2.7 (3)	3.6 (4)	97.3	**0.56** (0.11, 1.00)*
**Intersegmental mobility**					
Restriction	Cx	62.2 (69)	91.0 (101)	62.2	0.06 (-0.08, 0.19)
	Tx	18.9 (21)	48.7 (54)	61.3	0.21 (0.07, 0.36)
	Lx	52.3 (58)	46.9 (52)	62.2	0.25 (0.07, 0.43)
	SI	4.5 (5)	22.5 (25)	80.2	0.21 (0.02, 0.40)
Pain response	Cx	31.5 (35)	47.8 (53)	74.8	**0.49** (0.33, 0.64)
	Tx	12.6 (14)	27.0 (30)	82.0	**0.45** (0.26, 0.64)
	Lx	30.6 (34)	25.2 (28)	87.4	**0.69 (0.54, 0.84)**
	SI	5.4 (6)	8.1 (9)	95.5	**0.64** (0.36, 0.93)
**End range pain**					
Lumbar	R lat flex (2)	2.7 (3)	7.3 (8)	93.6	0.34 (-0.03, 0.70)*
	L lat flex (2)	4.6 (5)	8.2 (9)	92.7	0.39 (0.06, 0.73)
	Flex (1)	7.2 (8)	5.5 (6)	95.5	**0.59** (0.27, 0.92)
	Ext (4)	18.4 (20)	27.5 (30)	83.2	**0.54** (0.35, 0.72)
Cervical	R rot (1)	0.9 (1)	5.4 (6)	95.5	0.27 (-0.15, 0.70)*
	L rot (1)	0.9 (1)	4.5 (5)	96.4	0.32 (-0.16, 0.80)*
	Flex (2)	1.8 (2)	4.6 (5)	95.4	0.27 (-0.17, 0.71)*
	Ext (3)	4.5 (5)	2.8 (3)	94.4	0.22 (-0.18, 0.63)*

**P**_**a**_ = percentage of agreement; **Cx** = cervical spine; **Tx** = thoracic spine; **Lx** = lumbar spine; **SI** = sacroiliac joints; **R** = right; **L** = Left.

*The cell size of positive findings is ≤5, so the Kappa values should be interpreted with caution. Clinically acceptable Kappa values in bold.

Of the continuous variables (see Table 2), only FFD in forward flexion showed a high ICC [2.1] = 0.91 (95% CI: 0.87 - 0.94), whereas the other measures resulted in poor ICC values. For all variables, the LoA were wide, e.g. -9.0 - 7.6 for FFD in forward flexion compared with the range of assessments (0 – 32) (see Table 2). None of the Bland-Altman plots indicated heteroscedastic data.

**Table 2:** Inter-rater reliability and measurement error of commonly used clinical tests of the spine (continuous variables)

**Examination (missing values)**		**Measurement error**				**Reliability**
		**Range (cm)**	**đ (cm)**	**LoA (cm)**	**SEoM**	**ICC [2, 1] (95% CI)**
**General mobility**						
Lateral flexion FFD	R (1)	28.5, 57.5	2.56	-9.8, 15.0	4.4	0.47 (0.29, 0.62)
	L	32.5, 58.5	1.38	-9.1, 11.9	3.7	0.57 (0.43, 0.69)
Forward flexion FFD	(3)	0, 32.0	-0.72	-9.0, 7.6	2.9	**0.91 (0.87, 0.94)**

**Range** = lowest and highest measured value; **đ =** mean difference between ratings (systematic error); **LoA** = Limits of Agreement; **SEoM =** standard error of measurement; **CI** = confidence interval; **FFD** = finger-floor-distance; **R** = right; **L** = left; **ICC** = interclass correlation coefficient. Clinically acceptable ICC in bold.

### Intra-rater reliability

The results of the intra-rater reliability study are shown in Tables 3 and 4. In overall terms, intra-rater reliability was better than inter-rater reliability. Percentage agreement was ≥ 0.85 in 19 of the 27 tests for rater 1 and in 22 of the 27 tests for rater 2. Among the tests with cell sizes above five, we reached a Kappa value ≥ 0.4 for almost all variables with the exception of end range pain in lumbar flexion for rater 1 and shoulder height difference, inter-segmental restriction in the thoracic, and lumbar lateral flexion for rater 2. The cell size was small for many tests and should therefore be interpreted with caution. These tests are marked with a star in Table 3. For the continuous variables, only FFD in forward flexion resulted in a clinically acceptable ICC ≥ 0.75 for both raters. The measures of LoA were not satisfactory for any of the tests.

**Table 3:** Intra-rater reliability of commonly used clinical tests of the spine (binary variables)

**Test (missing values)**		**Prevalence of positive findings**				**Agreement**		**Reliability**	
		**Rater 1 (N = 39)**		**Rater 2 (N = 36)**		**Rater 1**	**Rater 2**	**Rater 1**	**Rater 2**
		**Ex 1% (n)**	**Ex 2% (n)**	**Ex 1% (n)**	**Ex 2% (n)**	**P**_**a **_**(%)**	**P**_**a **_**(%)**	**Kappa (95% CI)**	**Kappa (95% CI)**
**Scoliosis**									
Shoulder height		28.2 (11)	18.0 (7)	36.1 (13)	30.6 (11)	84.6	69.4	**0.59** (0.36, 0.85)	0.39 (0.37, 0.58)
Adam’s forward bend test (1)		12.8 (5)	18.0 (7)	11.4 (4)	5.6 (2)	84.6	94.3	**0.41** (0.03, 0.80)	**0.64** (0.19, 1.00)*
**Hypermobility**									
Knee	R	0.0 (0)	2.6 (1)	8.3 (3)	5.6 (2)	97.4	97.2	-	**0.79** (0.38, 1.00)*
	L	0.0 (0)	2.6 (1)	5.6 (2)	2.8 (1)	97.4	97.2	-	**0.65** (0.03, 1.00)*
Elbow	R	15.4 (6)	23.1 (9)	19.4 (7)	16.7 (6)	92.3	86.1	**0.76 (0.50, 1.00)**	**0.53** (0.17, 0.89)
	L	25.6 (10)	30.8 (12)	22.2 (8)	27.8 (10)	94.9	94.4	**0.87 (0.71, 1.00)**	**0.85 (0.66, 1.00)**
Thumb	R	10.3 (4)	10.3 (4)	13.9 (5)	13.9 (5)	100.0	94.4	1.00 (1.00, 1.00)*	**0.77 (0.46, 1.00)**
	L	12.8 (5)	10.3 (4)	8.3 (3)	11.1 (4)	92.3	97.2	**0.62** (0.24, 1.00)*	**0.84 (0.54, 1.00**)*
Fifth finger	R	0.0 (0)	0.0 (0)	2.8 (1)	0.0 (0)	-	-	-	-
	L	2.6 (1)	0.0 (0)	2.8 (1)	2.8 (1)	-	97.2	-	1.00 (1.00, 1.00)*
Trunk flexion (4)		0.0 (0)	0.0 (0)	2.9 (1)	2.9 (1)	97.4	100.0	-	1.00 (1.00, 1.00)*
Beighton score ≥ 4		2.6 (1)	5.1 (2)	5.6 (2)	5.6 (2)	97.4	100.0	**0.66** (0.03, 1.00)*	1.00 (1.00, 1.00)*
Beighton score ≥ 5		0.0 (0)	2.6 (1)	5.6 (2)	5.6 (2)	97.4	100.0	-	1.00 (1.00, 1.00)*
**Intersegmental mobility**									
Restriction	Cx	74.4 (29)	76.9 (30)	86.1 (31)	91.7 (33)	92.3	88.9	**0.79 (0.57, 1.00)**	**0.44** (-0.01,- 0.89)
	Tx	15.4 (6)	18.0 (7)	58.3 (21)	50.0 (18)	92.3	69.4	**0.72 (0.43, 1.00)**	0.39 (0.09, 0.69)
	Lx	38.5 (15)	56.4 (22)	47.2 (17)	41.7 (15)	71.8	77.8	**0.45** (0.19, 0.71)	**0.55** (0.28, 0.82)
	SI	2.6 (1)	2.6 (1)	22.2 (8)	11.1 (4)	94.9	83.3	-0.03 (-0.09, 0.04)*	**0.41** (0.04, 0.78)*
Pain response	Cx	35.9 (14)	33.3 (13)	44.4 (16)	38.9 (14)	87.2	88.9	**0.72 (0.49, 0.95)**	**0.77 (0.56, 0.98)**
	Tx	12.8 (5)	18.0 (7)	33.3 (12)	38.9 (14)	94.9	83.3	**0.80 (0.55, 1.00)**	**0.64** (0.38, 0.90)
	Lx	25.6 (10)	33.3 (13)	27.8 (10)	38.9 (14)	92.3	88.9	**0.82 (0.62, 1.00)**	**0.75 (0.53, 0.97)**
	SI	5.1 (2)	2.6 (1)	8.3 (3)	8.3 (3)	92.3	94.4	-0.04 (-0.11, 0.04)*	**0.64** (0.17, 1.00)*
**End range pain**									
Lumbar	R lat flex (2)	5.1 (2)	5.1 (2)	2.9 (1)	8.6 (3)	100.0	91.2	1.00 (1.00, 1.00)*	0.00 ( - , 1.00)*
	L lat flex (2)	12.8 (5)	5.1 (2)	2.9 (1)	8.6 (3)	92.3	88.2	**0.54** (0.09, 0.98)*	-0.05 (-0.14, 0.05)*
	Flex	18.0 (7)	12.8 (5)	2.8 (1)	5.6 (2)	79.5	91.7	0.22 (-0.16, 0.60)	-0.04 (-0.12, 0.04)*
	Ext (4)	23.7 (9)	23.7 (9)	34.3 (12)	25.7 (9)	94.6	82.4	**0.85 (0.66, 1.00)**	**0.58** (0.28, 0.88)
Cervical	R Rot (1)	0.0 (0)	0.0 (0)	5.6 (2)	2.8 (1)	-	94.4	-	0.00 ( - , 1.00)*
	L Rot (1)	0.0 (0)	0.0 (0)	5.6 (2)	0.0 (0)	-	91.7	-	-0.04 (-0.12, 0.04)*
	Flex (1)	5.3 (2)	5.1 (2)	5.6 (2)	2.8 (1)	100.0	97.2	1.00 (1.00, 1.00)*	**0.65** (0.03, 1.00)*
	Ext (1)	2.6 (1)	5.1 (2)	5.6 (2)	5.6 (2)	97.4	94.4	0.66 (0.03, 1.00)*	**0.47** (-0.15, 1.00)*

**Ex** = examination; **P**_**a**_ = percentage of agreement; **Cx** = cervical spine; **Tx** = thoracic spine; **Lx** = lumbar spine; **SI** = sacroiliac joints; **R** = right; **L** = left; **ROM** = Range of motion.

*The cell size of positive findings is ≤5, so the Kappa values should be interpreted with caution. Clinically acceptable Kappa values in bold.

**Table 4:** Intra-rater reliability and measurement error of commonly used clinical tests of the spine (continuous variables)

**Examination (missing values)**			**Measurement error**				**Reliability**
		**Rater**	**Range (cm)**	**đ (cm)**	**LoA (cm)**	**SEoM**	**ICC [2, 1] (95% CI)**
**General mobility**							
Lateral flexion FFD	R (1)	1	29.5, 58.0	1.53	-7.5, 10.5	3.3	**0.77** (0.60, 0.88)
	R	2	36.0, 51.5	1.00	-5.7, 7.7	2.4	0.65 (0.41, 0.80)
	L	1	34.0, 57.0	0.56	-6.2, 7.3	2.4	**0.84** (0.72, 0.91)
	L	2	34.5, 52.0	0.33	-6.7, 7.3	2.4	0.64 (0.40, 0.80)
Forward flexion FFD	(1)	1	0, 34.5	-0.77	-10.3, 8.7	3.4	**0.89 (0.80, 0.94)**
	(3)	2	0, 31.5	-0.06	-6.7, 6.6	2.3	**0.94 (0.89, 0.97)**
Schober test		1*	13.3, 15.5	0.03	-1.3, 1.3	0.4	0.53 (0.26, 0.73)
		2*	13.0, 16.0	-0.03	-2.9, 2.8	1.0	0.14 (-0.21, 0.45)

**Range** = lowest and highest measured value; **đ =** mean difference between ratings (systematic error); **LoA** = Limits of Agreement; **SEoM =** standard error of measurement; **CI** = confidence interval; **FFD** = finger-floor-distance; **R** = right; **L** = left; **ICC** = interclass correlation coefficient. *In the Schober test rater 1 rounded up/down to nearest half cm, rater 2 by fault to nearest whole cm. Clinically acceptable ICC in bold.

## Discussion

In summary, based on the predefined cut points for Kappa and ICC values, clinically acceptable inter-rater reliability was found for most hypermobility tests, inter-segmental mobility with pain response and lumbar end range pain in flexion and extension. Results for FFD in forward flexion were difficult to interpret and all other test variables showed poor to slight Kappa values or unacceptable ICCs and LoA of inter-rater assessment. The intra-rater values were on average in the middle between the inter-rater values and 1.00 so there are discrepancies both between the observers and at the single observer level.

The two tests for *scoliosis* did not perform well in our study with slight inter-rater reliability and moderate intra-rater reliability with very wide CI. The examiners, however, did not report any contradictory results, where one assessed a higher left and the other a higher right shoulder (data not shown). To our knowledge, no comparable studies have investigated the reliability of the shoulder height difference test. One study evaluated the reliability of Adam’s Forward Bend Test in a population already defined with scoliosis [10]. They reported a Kappa value somewhat higher than in the current study; however this is likely to be explained by the difference in population in the study. The prevalence rate was in excess of 70%, whereas in our study population, the prevalence rate was around 10%, resulting in small cell sizes and thus less trustworthy Kappa values. The poor result for the Adam’s Forward Bend Test might also have been due to the subjects wearing a shirt during the assessment.

Most of the tests for *hypermobility* were reliable. The exceptions were tests for knee and fifth finger extension. It should be noted that the prevalence of positive findings for these two conditions were very low with cell sizes ranging from 1 to 10 which could contribute to the low Kappa values. Knee hyperextension was probably influenced by the subjects wearing pants while being assessed. Earlier studies have shown good reliability when the tests were evaluated as a whole (index sum score) but these studies were either performed on adults or used other statistical methods [11-14]. Since the cut-off level for a positive index score is debatable [28], we calculated reliability using the Beighton score with cut-off points at both 4 and 5, and found moderate to almost perfect agreement for both, which is in line with another study using the same cut-off points [14]. However, we observed that even if the raters agreed with a cut-off point at 5, they did not necessarily agree on which of the joints were included in this score, e.g. one elbow, two thumbs and two fifth fingers were compared to two knees, two elbows and two fifth fingers in the inter-rater reliability study; that means they only agreed in three of the nine joints, but both had a Beighton score ≥ 5. Although this might discredit the individual tests, it shows that the index score is robust.

Some studies demonstrated excellent inter- and intra-rater reliability for FFD both for *forward and lateral flexion*[15-18]. However, these studies used adult subjects and furthermore, a different approach in performing the tests and/or the statistical measures was applied. The inter-rater reliability of FFD in forward flexion was also high in our study, indicating an ability of this test to distinguish subjects from each other. However, the LoA were wide compared with the range of assessments, implying that the scores of repeated measurements differed substantially. Because ICC is affected by the total variance [32], a high variance in our subject population could somewhat obscure the measurement error in the ICC value, explaining why a clinically unacceptable LoA is accompanied by a high ICC. This means that the positive results should be interpreted with caution.

The poor reliability and large measurement error in lateral flexion in our study might be related to the difficulty in performing a pure spinal lateral flexion. We suggest modifying this test to have the subject standing against a wall during the assessment, as performed in another study [18]. This would probably reduce the negative influence of combined flexion/extension or rotation with lateral flexion on measurement accuracy, as was observed in many cases.

Regarding *the Schober test*, a study using a similar approach in adult men with known ankylosing spondylitis has shown excellent reliability: ICC = 0.93 and 0.96 for inter- and intra-rater reliability respectively [16]. For intra-rater reliability and measurement error, we see that both ICC and LoA differ substantially between the raters, probably because of the mistake made by Rater 2 who rounded up or down to the nearest whole cm e.g. LoA was calculated to be -1.3 – 1.3 for Rater 1 vs. -2.9 - 2.8 for Rater 2. We believe that the results in our study may also have been negatively influenced by a slight variation in starting position due to lack of agreement in how to locate the bony landmarks, a difficulty described elsewhere [33].

Assessment for *inter-segmental mobility* showed better reliability when assessing for pain than when assessing for restricted movement. In general, the intra-rater reliability was higher than the inter-rater reliability. This is consistent with an earlier review [19].

The outcome for *end range pain* in the cervical and lumbar spine showed inconsistent results. Again, better results were seen with intra-rater assessment. An earlier study evaluating inter-rater reliability for end range pain in the cervical spine also demonstrated high variability of results and unacceptable ICCs for most of the variables [20]. Lumbar pain in extension scored the highest Kappa values, but the pain in this position was relatively common, reaching a prevalence of 28% (cell size: 30), while a maximum of 8% (cell size: 9) was reported for pain in the other lumbar and cervical movement directions. This fact could partly explain the difference in Kappa values. However, there were some difficulties and discrepancies connected with the performance of these tests, which also led to decreased reliability. The observers noted frequent uncertainty when classifying the responses. Some subjects used the term “soreness”, which was interpreted differently between the raters. We believe that there is more to be gained with refined practice and further standardisation of these tests.

The study’s main strength is its school-based population which reflects the target population, i.e. the age where the prevalence of spinal pain escalates. In addition, we nearly reached a 100% participation rate with an almost equal distribution between genders, minimising possible bias due to gender disproportion.

The pre-study training session, the presence of the observers and our use of two well-experienced chiropractors as raters could have contributed to the relatively high reliability measures of this study. One could argue that this is not representative of the true situation in a school screening setting, however, we think the tests are easy to perform, the interpretation relatively easy (“yes”/“no”) and that the tests do not need special skills except for the inter-segmental mobility, where long term experience is beneficial. One would assume after a few training sessions that the tests could be used by any practitioner dealing with spinal examinations.

The major limitation of our study is the sample size. Although we exceeded our goal of 100 subjects, the prevalence rates of a positive test were very low for some of the tests. We believe a larger sample size and thus more positive findings would result in more precise reliability estimates, which means either higher or lower than estimated in this study. Despite the standardization of the tests, the observers occasionally noted discrepencies in the instructions to perform a maximal forward flexion. This could have resulted in less precise estimates of ICC and measurement error of forward flexion finger-floor-distance and the Schober test if the protocol was followed. On the other hand, the same would probably occur in a screening setting at schools or in the clinics and therefore probably gives a more realistic estimate of the test’s reliability and measurement error. The relatively short time period between examinations in the intra-rater part of the study could also be a limitation by increasing the risk of raters recalling an individual’s test results from an earlier examination. However, we judged this influence to be less detrimental than the potential risk of changes in the subjects’ biomechanical state arising as a result of a longer interval between the two examinations where injuries and/or new onsets of spinal pain could occur. Furthermore, the large battery of tests in the protocol and the many subjects assessed in between the first and the second examination of each subject is deemed to have minimised the risk of memory bias. The time limitation could also have been a factor that negatively affected our results. The raters, however, believed that their performance would not have been any different under other time conditions, as they seldom needed the 4 minutes to complete a single examination. We therefore consider this time consideration to be of minor relevance.

## Conclusion

Some clinical tests showed good, and some tests poor, reliability when applied in a spinal screening of adolescents. Acceptable reliability was found for Beighton index score for hypermobility, inter-segmental mobility with pain response and lumbar end range pain in flexion and extension and we believe these tests can be performed reliably by clinicians with relevant experience. Results of FFD in forward flexion were difficult to interpret. The results could probably be improved by additional training and further test standardisation. This is the first step in evaluating the value of these tests for the spinal screening of adolescents. Future research should determine the association between these tests and current and/or future neck and back pain.

## Appendix 1. Test variables

## Assessment of scoliosis

## Shoulder height difference

With the subject standing upright, an observed difference in shoulder height was noted.

## Adam’s forward bend test

The subject was standing in an upright position and asked to flex forward while the rater looked for trunk asymmetry along the horizontal plane, known as a “rib hump”, which was considered a positive finding. This test was conducted in conjunction with the Schober test.

## Assessment of hypermobility

In assessing hypermobility, the tests included in Beighton Joint Mobility Index were used [22].

## Knee extension

With the subject standing upright, knee hyperextension greater than 10 degrees was considered a positive finding. A slight pressure from raters was used to achieve end-range extension.

## Elbow extension

While stabilising the distal part of the forearm, a gentle force was applied from the posterior side of the elbow joint, to achieve passive end-range extension. Hyperextension greater than 10 degrees was considered a positive test.

## Thumb abduction/opposition and wrist flexion

With the subject’s wrist in flexion, the thumb was passively abducted. The subject was then asked to approximate the thumb to the volar part of the forearm. Contact was considered a positive test.

## Fifth finger extension

While stabilising the fifth metacarpal, the fifth finger was passively extended as far as possible without pain. Extension greater than 90 degrees was considered a positive test.

## Trunk and hip flexion

The subject was standing upright and asked to flex forward as far as possible with their knees fully extended. If the subject was able to touch the ground with the palms of both hands, the test was considered positive. This test was performed in conjunction with the forward flexion test for general mobility.

## Assessment of general mobility

## Forward flexion

The subject was standing erect and was asked to flex forward as far as possible with knees fully extended. The distance between the fingertips and floor (FFD = Finger-Floor-Distance) was measured in cm.

## Lateral flexion

The subject was standing upright and asked to laterally bend their spine while letting the hand slide down the leg. If forward flexion of the spine or hip occurred, this was corrected. FFD was measured in cm on both sides.

## The Schober test

The posterior superior iliac spines (PSIS) were located and a point between them was marked with a sticker. Another point 10 cm superior to this was fixed with a tape measure. The subject was then asked to bend forward as far as possible and the distance between the fixed point and the sticker was measured to the nearest half cm.

## Inter-segmental mobility

## Restriction

The subject was seated upright while the rater manually palpated the segmental movement between adjacent vertebrae in the cervical, thoracic and lumbar regions and between the sacral and iliac bones. At end range, a light pressure was applied on the spinous process, the facet joints or SI joints, to evaluate the quality of movement and end feel. Restricted segmental movement on at least one segment in the cervical, thoracic, or lumbar spinal regions and/or SI joints was noted as a positive finding for the respective region.

## Pain response

Pain response due to the light pressure applied on the spinous process, facet joints or SI joints was noted for each spinal region. The examiner inquired about pain during the examination.

## End range pain on active range of motion

## Pain in maximal lumbar flexion

When measuring FFD in flexion, the subject was asked if pain was experienced and, if applicable, where the pain was located. A pain response located in the lumbar spine was noted as a positive test.

## Pain in maximal lumbar lateral flexion

When measuring FFD in lateral flexion, the subject was asked if pain was experienced and, if applicable, where the pain was located. Pain located in the lumbar spine was noted as a positive test. Contralateral pain was interpreted as muscle stretch, and ipsilateral pain located more laterally was considered to be compression pain between lower ribs and pelvis, and therefore both were considered a negative test.

## Pain in maximal lumbar extension

The subject was standing upright. The rater placed one hand on the patient’s sacrum and the other one on the patient’s chest/shoulder to induce the movement. The subject was then asked to perform a maximal lumbar extension. The subject was asked if pain was felt, and where it was located. Pain located in the lumbar spine was noted as a positive test.

## Pain in cervical end range of motion

The subject was seated upright and was asked to move the neck into maximum flexion, extension, and right/left rotation. The rater was allowed to gently induce the movement as part of the instruction. The subject was asked if pain was experienced. Pain located in the cervical spine was noted as a positive test.

## Competing interests

The authors declare that they have no competing interests.

## Authors’ contributions

EA designed the study, contributed to the data collection, did the data analyses and wrote the manuscript. AD and LK contributed to the data collection and the writing of the manuscript. LH contributed to the selection of the tests, the pilot study and the writing of the manuscript. All authors read and approved the final manuscript.

## Pre-publication history

The pre-publication history for this paper can be accessed here:

http://www.biomedcentral.com/1471-2474/15/37/prepub
